# Cell-free hemoglobin mediated oxidative stress is associated with acute kidney injury and renal replacement therapy in severe falciparum malaria: an observational study

**DOI:** 10.1186/s12879-017-2373-1

**Published:** 2017-04-27

**Authors:** Katherine Plewes, Hugh W.F. Kingston, Aniruddha Ghose, Richard J. Maude, M. Trent Herdman, Stije J. Leopold, Haruhiko Ishioka, Md. Mahtab Uddin Hasan, Md. Shafiul Haider, Shamsul Alam, Kim A. Piera, Prakaykaew Charunwatthana, Kamolrat Silamut, Tsin W. Yeo, Md. Abul Faiz, Sue J Lee, Mavuto Mukaka, Gareth D.H. Turner, Nicholas M. Anstey, L. Jackson Roberts, Nicholas J. White, Nicholas P.J. Day, Md. Amir Hossain, Arjen M. Dondorp

**Affiliations:** 1 Mahidol Oxford Tropical Medicine Research Unit, Faculty of Tropical Medicine, Mahidol University, Bangkok, Thailand; 2 Centre for Tropical Medicine and Global Health, Nuffield Department of Medicine, University of Oxford, Oxford, UK; 3 Department of Medicine and Vancouver Coastal Health, University of British Columbia Clinical Investigator program, Vancouver, Canada; 4 Global Health Division, Menzies School of Health Research and Charles Darwin University, Darwin, Northern Territory Australia; 5 Department of Medicine, Chittagong Medical College and Hospital, Chittagong, Bangladesh; 6 Department of Nephrology, Chittagong Medical College and Hospital, Chittagong, Bangladesh; 7 Department of Anesthesiology, Chittagong Medical College and Hospital, Chittagong, Bangladesh; 8 Lee Kong Chian School of Medicine, Nanyang Technological University, Singapore, Singapore; 9 Dev Care Foundation, Dhaka, Bangladesh; 10 Department of Pharmacology, Vanderbilt University, Nashville, TN USA

**Keywords:** Acute kidney injury, Pathophysiology, Falciparum malaria, Cell-free hemoglobin, Oxidative stress

## Abstract

**Background:**

Intravascular hemolysis is an intrinsic feature of severe malaria pathophysiology but the pathogenic role of cell-free hemoglobin-mediated oxidative stress in severe malaria associated acute kidney injury (AKI) is unknown.

**Methods:**

As part of a prospective observational study, enrolment plasma cell-free hemoglobin (CFH), lipid peroxidation markers (F_2_-isoprostanes (F_2_-IsoPs) and isofurans (IsoFs)), red cell deformability, and serum creatinine were quantified in Bangladeshi patients with severe falciparum malaria (*n* = 107), uncomplicated malaria (*n* = 80) and sepsis (*n* = 28). The relationships between these indices and kidney function and clinical outcomes were examined.

**Results:**

AKI was diagnosed at enrolment in 58% (62/107) of consecutive patients with severe malaria, defined by an increase in creatinine ≥1.5 times expected baseline. Severe malaria patients with AKI had significantly higher plasma cell-free hemoglobin (geometric mean CFH: 8.8 μM; 95% CI, 6.2–12.3 μM), F_2_-isoprostane (56.7 pg/ml; 95% CI, 45.3–71.0 pg/ml) and isofuran (109.2 pg/ml; 95% CI, 85.1–140.1 pg/ml) concentrations on enrolment compared to those without AKI (CFH: 5.1 μM; 95% CI, 4.0–6.6 μM; *P* = 0.018; F_2_-IsoPs: 27.8 pg/ml; 95% CI, 23.7–32.7 pg/ml; *P* < 0.001; IsoFs: 41.7 pg/ml; 95% CI, 30.2–57.6 pg/ml; *P* < 0.001). Cell-free hemoglobin correlated with markers of hemolysis, parasite burden (*P. falciparum* histidine rich protein 2 (PfHRP2)), and F_2_-IsoPs. Plasma F_2_-IsoPs and IsoFs inversely correlated with pH, positively correlated with creatinine, PfHRP2 and fractional excretion of sodium, and were higher in patients later requiring hemodialysis. Plasma F_2_-IsoP concentrations also inversely correlated with red cell deformability and were higher in fatal cases. Mixed effects modeling including an interaction term for CFH and time showed that F_2_-IsoPs, IsoFs, PfHRP2, CFH, and red cell rigidity were independently associated with increasing creatinine over 72 h. Multivariable logistic regression showed that admission F_2_-IsoPs, IsoFs and red cell deformability were associated with the need for subsequent hemodialysis.

**Conclusions:**

Cell-free hemoglobin and lipid peroxidation are associated with acute kidney injury and disease severity in falciparum malaria, suggesting a pathophysiological role in renal tubular injury. Evaluation of adjunctive therapies targeting cell-free hemoglobin-mediated oxidative stress is warranted.

**Electronic supplementary material:**

The online version of this article (doi:10.1186/s12879-017-2373-1) contains supplementary material, which is available to authorized users.

## Background

Severe falciparum malaria is characterized by intravascular hemolysis, where cell-free hemoglobin (CFH) increases with disease severity [1]. Sources of CFH include rupture of parasitized red blood cells (RBC) at schizogony, and destruction of uninfected erythrocytes, most prominently in patients with blackwater fever (hemoglobinuria) [2]. In 400 BC, Hippocrates first associated blackwater fever with anuria and mortality; findings that consistently resurfaced after Firth’s report in 1886 [3, 4]. More recently, this condition of fulminant hemolysis has been associated with kidney dysfunction in up to 64% of patients [5], but the underlying mechanisms have not been fully characterized.

When the degree of intravascular hemolysis exceeds the scavenging capacity of plasma haptoglobin for hemoglobin, CFH dimers are filtered by the glomeruli and reabsorbed by the proximal tubule. Once the reabsorptive capacity is exceeded, hemoglobin appears in the urine [6]. CFH is independently associated with AKI in patients post-cardiopulmonary bypass, and with mortality in bacterial sepsis [7–9]. Hemoproteins, hemoglobin and myoglobin, are pathogenic as pro-oxidants when released heme is not scavenged by hemopexin. Heme redox cycling between ferric and ferryl states then generates globin radicals inducing lipid peroxidation [10]. In vivo studies on oxidative injury have been hampered by the paucity of stable and specific markers of oxidative stress.

CFH-mediated non-enzymatic lipid peroxidation of arachidonic acid generates isomers of prostaglandins, F_2_-isoprostanes (F_2_-IsoPs) and isofurans (IsoFs) [11, 12]. F_2_-IsoPs are generated at low oxygen tension whereas IsoFs are generated at higher oxygen tension and together are considered robust in vivo measures of oxidative stress [11, 12]. In the current study, the hypothesis was that CFH-mediated oxidative stress could cause renal damage either through a direct effect on renal tubules, through a reduction in red cell deformability (RCD), or through the vasoconstrictive properties of F_2_-IsoPs.

Arachidonic acids, such as red cell membrane phospholipids, are particularly vulnerable to free radical-mediated lipid peroxidation. Oxidative stress-induced reduction of RCD has been proposed to play a role in renal insufficiency [13]. In malaria, the high arachidonic acid content of infected RBC membranes reduces with intracellular parasite maturation, suggesting membrane peroxidation occurs during parasite development [14]. F_2_-IsoPs are considered not just bystanders of oxidative injury but are bioactive renal vasoconstrictors [12]. Both F_2_-IsoPs and IsoFs have been associated with AKI in patients with rhabdomyolysis and hemolysis post-cardiopulmonary bypass [15–17]. Other plasma and urinary markers of oxidative stress have been shown to be significantly elevated in severe malaria compared to uncomplicated malaria [18–21].

The role of plasma CFH-mediated lipid peroxidation in the pathophysiology of severe malaria and AKI has not been described. Gaining a better understanding of the pathophysiology in malaria will help towards the development of targeted therapies. This study aimed to examine the generation of CFH-mediated lipid peroxidation and its role in AKI and malaria severity by analyzing the associations between CFH, F_2_-IsoPs, IsoFs, red cell deformability, and creatinine in patients with falciparum malaria.

## Methods

### Study aim, design and setting

The aim of this study was to assess CFH-mediated lipid peroxidation and its role in AKI and disease severity in falciparum malaria. This prospective observational study was conducted at Chittagong Medical College Hospital, Bangladesh from 2011 to 2014. This tertiary hospital receives referrals from malaria hypoendemic areas, and has basic facilities for intensive care and hemodialysis.

### Patient characteristics

Patients admitted with slide confirmed severe or uncomplicated *P. falciparum* malaria were recruited upon diagnosis. Positive microscopy of peripheral blood required the presence of asexual stages of *P. falciparum*. Uncomplicated malaria was defined as asexual *P. falciparum* slide positivity without severity criteria. Criteria for severe malaria were: coma (Glasgow Coma Score < 11), shock (systolic blood pressure (SBP) < 80 mmHg with cool extremities), anemia, (hematocrit <20% plus parasitemia >100,000/μl), jaundice (total bilirubin >51.3 μmol/L plus parasitemia >100,000/μl), hyperparasitemia (asexual parasitemia >10%), acidosis (bicarbonate <15 mmol/L), hyperlactatemia (lactate >4 mmol/L), hypoglycemia (glucose <2.22 mmol/L), convulsions (≥ 2 in 24 h), pulmonary edema, and/or AKI (serum creatinine >3 mg/dl). Patients were treated with parenteral artesunate (Guilin No.2 Pharmaceuticals, Guangxi, China) followed by artemether/lumefantrine (Novartis, Basel, Switzerland) and managed according to WHO treatment guidelines [22]. Hemodialysis was initiated according to local nephrologists. Indications for dialysis in this setting include: (1) anuria for more than 24 h, (2) severe electrolyte and acid-base disturbance, (3) serum creatinine >3 mg/dl with urine output <0.5 ml/kg/h for 12 h, or (4) gradual rise in creatinine despite normal urine output. A control group of sepsis patients hospitalized with suspected bacterial infection and at least 2/4 systemic inflammatory response criteria (*n* = 28) was also recruited [23]. Data from non-pregnant patients aged ≥10 years is presented. Patients were followed until discharge or death; those with AKI were followed in hospital until renal recovery, if possible, as gauged by local nephrologists. Follow up after hospital discharge was challenging due to the distances patients travel to the teriary care center.

### Study procedures

After enrolment, a medical history and physical examination were performed. Patients were seen 6-hourly until discharge or death. Enrolment venous blood samples were analyzed for electrolytes, glucose, pH and bicarbonate using a bedside analyzer (iSTAT, Abbott). Parasitemia was assessed 6-hourly from thick and thin smears until parasite clearance. Blood and urine for creatinine measurement were collected every 24 h for three days. Serum, heparinized plasma and urine were frozen in liquid nitrogen within one hour of collection. Serum creatinine was measured using an Olympus analyzer (Beckman Coulter Inc.).

### Assays

Plasma and urine F_2_-isoprostane and isofuran concentrations were determined by gas chromatography-mass spectrometry at Vanderbilt University, as described [11, 24]. The 24-h urinary excretion rate of urine F_2_-isoprostane and isofuran concentrations were calculated as: concentration x volume × 24 h/time of collection [25]. Plasma CFH concentrations were measured by ELISA (Bethyl Laboratories), as described [1]. Plasma *Plasmodium falciparum* histidine rich protein 2 (PfHRP2), a biomarker of total parasite burden, was quantified using commercial sandwich ELISA (Celisa, Cellabs), as described [26].

Red cell deformability (RCD) was measured at enrolment using a laser-assisted optical rotational cell analyzer (LORCA, Mechatronic) immediately after blood collection [27, 28]. The deformability was measured by ellipticity of RBCs and described by the elongation index; (long minus short axes lengths) divided by (long plus short axes lengths). RCD was assessed at shear stresses ranging from 0.3 to 30 Pa. In capillaries, shear stresses of 1.7 Pa and above are encountered [29].

### Acute kidney injury

Patients were classified according to AKI status at enrolment as defined by an increase in serum creatinine ≥1.5 times expected baseline, known or presumed to have occurred within the prior seven days (as per the Kidney Disease Improving Global Outcomes (KDIGO) classification system) [30]. Since urine output was not available for all patients, and the duration of illness at presentation was always greater than 48 h, these criteria were not incorporated for enrolment AKI diagnosis. As pre-admission creatinine values were not available, expected baseline creatinine values were calculated as recommended using the Modification of Diet in Renal Disease formula assuming a glomerular filtration rate (GFR) of 75 ml/min/1.73m^2^ for participants 19 years and older [30] and using the Bedside Schwartz formula assuming a GFR of 100 ml/min/1.73m^2^ for those 18 years and younger [31–33]. The highest KDIGO AKI staging was assessed both on enrolment and during admission in order to accurately present the heterogeneous AKI status at the time of enrolment and subsequent kidney function during admission in those that survived. Stage 2 AKI was defined as an increase to ≥ 2.0–2.9 times expected baseline; Stage 3 as either an increase to ≥ 3 times expected baseline, an increase in creatinine to ≥4 mg/dl, initiation of RRT, or in patients <18 years a decrease in GFR to <35 ml/min/1.73 m^2^ [30]. Creatinine used for enrolment AKI stratification were performed on samples drawn prior to hemodialysis.

### Statistical analysis

Groups were compared using Wilcoxon rank-sum test or Student’s *t*-tests depending on the distribution of the data. Correlations were assessed using Spearman’s correlation coefficient. As the hypothesis was that F_2_-IsoPs, IsoFs, and CFH contribute to AKI, these pre-specified variables were assessed in a mixed effects model using creatinine as the dependent variable, and in a logistic regression model using hemodialysis as the dependant variable. As the F_2_-IsoPs and IsoFs were highly collinear (*r*_*p*_ = 0.67; *p* < 0.001), their association with creatinine was assessed in separate multivariable models. The multivariable models included adjustment for age, SBP, PfHRP2 and RCD as these are considered physiologically relevant in contributing to AKI. In the mixed effect model, age, SBP, and time were modeled as fixed effects while the rest were treated as random effects. All pre-specified and known AKI risk factor variables that were associated with creatinine were included in a multivariable mixed effects model. The interaction between enrolment CFH and time was also assessed. To account for hemodialysis confounding the decline in creatinine concentrations, creatinine values were adjusted at each time point following dialysis until time of death by using a creatinine rise of 1.5 mg/dl per day as proposed for anephric states [34]. Known risk factors for hemodialysis, including additional markers of disease severity (number of severity criteria, GCS, and lactate), were assessed using backward stepwise logistic regression including variables (Table 5) with a *p*-value of <0.10 on univariable analysis. Selection of the final model was based on the Akaike information criteria (AIC). Software used were STATA14.0 (Stata), and Prism 6 (Graphpad Software).

## Results

### Baseline characteristics and clinical course in hospital

A total of 107 consecutive patients with severe malaria were enrolled (Tables 1 and 2), as well as 80 patients with uncomplicated malaria and 28 with (suspected) bacterial sepsis as comparator control groups. Among patients with severe malaria, 58% (62/107) had AKI on enrolment, while another 9% (10/107) subsequently developed AKI during admission (Table 1). The severity of AKI varied with 50% (31/62) meeting the World Health Organization malaria guideline criteria for AKI (creatinine >3 mg/dl) [22], of whom 84% (26/31) were KDIGO stage 3 and 16% (5/31) had a further progression from KDIGO stage 2 to 3 during admission. In the AKI on enrolment group, 47% (29/62) received hemodialysis; of whom 28% (8/29) died, and 53% (33/62) did not receive hemodialysis; of whom 52% (17/33) died (OR for death without dialysis 2.8 (95% CI, 0.9–9.4; *P* = 0.048). Among the 72% (21/29) survivors in the AKI group who received dialysis, the median renal recovery time was 21 days (IQR, 13–42 days; *n* = 7). Among the 48% (16/33) survivors in the AKI group who did not receive dialysis, the median renal recovery time was three days (IQR, 2–4 days; *n* = 14). Of 10/107 patients who developed AKI after admission, 30% (3/10) received hemodialysis and 40% (4/10) died, compared to an overall mortality in the severe malaria cohort of 33% (35/107) (Table 3). The mortality rate among all patients admitted with or developing AKI but not receiving dialysis was nearly double (20/40; 50%) that of patients to those who did (9/32; 28%)(OR 2.6, 95% CI, 0.9–7.8; *P* = 0.09). Those with AKI on enrolment had more severe disease, as defined by a higher median number of WHO severity criteria (*P* < 0.001). No patient reported a history of kidney disease and there was no difference in comorbidities (hypertension, diabetes and cardiovascular disease) between groups (Table 1).

**Table 1:** Baseline demographics and clinical characteristics of patients with severe falciparum malaria by AKI status at enrolment

Variable	Total	No AKI	AKI	*P*
	(*n* = 107)	(*n* = 45)	(*n* = 62)	
Demographics				
Age (years)	30 (22–40)	30 (25–45)	27 (18–40)	0.104
Males (%)^c^	75 (70)	35 (78)	40 (65)	0.200
Fever prior to admission (days)	7 (6–9)	7 (6–8)	7 (6–9)	0.311
History of black or red urine^c^	20 (20)	5 (11)	15 (26)	0.100
Vomiting and diarrhea (days)	5 (1–6)	2.5 (1–5)	5 (3–9)	0.045
Comorbidities				
Hypertension^c^	5 (5)	2 (4)	3 (5)	1.000
Cardiovascular disease^c^	6 (6)	2 (4)	4 (6)	1.000
Type 2 diabetes^c^	4 (4)	3 (7)	1 (2)	0.307
Enrolment clinical parameters				
Glasgow Coma Score (max 15)	9 (8–14)	10 (9–14)	9 (7–14)	0.354
Systolic blood pressure (mmHg)	110 (100–120)	114 (102–120)	107 (99–120)	0.155
Mean arterial pressure (mmHg)	82 (72–90)	81 (74–89)	82 (71–94)	0.550
Pulse rate (breaths/min)	115 (96–132)	109 (93–131)	116 (97–132)	0.666
Respiratory rate (breaths/min)	34 (28–42)	32 (28–37)	36 (28–44)	0.370
Hemoglobinuria on enrolment^# c^	18 (17)	4 (9)	14 (24)	0.124
Number of severity criteria	2 (1–3)	1 (1–2)	2 (2–4)	<0.001
AKI stage at enrolment				
Stage 1 (≥1.5 × baseline)^c^	16 (26)	--	16 (26)	--
Stage 2 (≥2.0–2.9 × baseline)^c^	16 (26)	--	16 (26)	--
Stage 3 (≥3.0 × baseline or ≥4 mg/dl)^c^	30 (48)	--	30 (48)	--

All values are median (IQR) unless otherwise specified: ^c^number (%). *P* < 0.05 using student t-test or Mann-Whitney U. # hemoglobinuria defined as red, black or dark brown urine on exam with 3/4+ hemoglobin on urine dipstick. *Abbreviations: AKI* acute kidney injury

**Table 2:** Baseline admission laboratory parameters of patients with severe falciparum malaria by AKI status at enrolment

Variable	Total	No AKI		AKI		*P*	
	(*n* = 107)	n	(*n* = 45)	n	(*n* = 62)	n	
Hemoglobin (mg/dl)	9.1 (7.2–11.0)	107	10.6 (8.0–11.3)	45	8.5 (7.1–10.3)	62	0.017
Cell-free hemoglobin (μM)^b^	7.0 (5.5–8.7)	105	5.1 (4.0–6.6)	45	8.8 (6.2–12.3)	60	0.018
White blood cells (×10^3^/μl)	9.4 (6.7–12.8)	102	9.2 (6.4–11.7)	44	9.6 (7.0–16.6)	58	0.339
Platelets (×10^3^/μl)	30 (19–46)	100	35 (23–53)	44	27 (18–42)	56	0.075
Total bilirubin (mg/dl)	2.0 (1.0–5.3)	106	1.5 (0.9–3.2)	45	2.5 (1.3–10.7)	61	0.005
Indirect bilirubin (mg/dl)	0.8 (0.3–1.9)	106	0.4 (0.2–1.3)	45	1.3 (0.4–2.7)	61	0.013
Lactate dehydrogenase (U/l)	635 (455–886)	107	541(403–643)	45	766 (566–1027)	62	<0.001
Creatinine (mg/dl)	1.4 (1.1–3.3)	107	1.2 (1.0–1.3)	45	3.0 (1.6–4.4)	62	<0.001
Blood urea nitrogen (mg/dl)	43 (26–75)	107	26 (18–37)	45	66 (44–104)	62	<0.001
Potassium (mmol/l)	4.4 (3.9–5.2)	106	4.1 (3.8–4.6)	45	4.7 (4.1–5.5)	61	<0.001
Base excess (mmol/l)	−8 (−11 to −4)	107	−5 (−7 to −2)	45	−10 (−13 to −7)	62	<0.001
Bicarbonate (mmol/l)	17.3 (14.1–20.2)	107	19.2 (17.0–21.9)	45	15.9 (13.3–18.6)	62	<0.001
Lactate (mmol/l)	3.85 (2.49–6.28)	107	3.85 (2.58–5.61)	45	3.89 (2.30–6.68)	62	0.852
Parasitemia (parasites/μl)^b^	53,529 (34586–82,846)	107	61,253 (34094–110,048)	45	48,540 (25716–91,619)	62	0.605
PfHRP2 (ng/ml)	2584 (1341–7194)	101	1743.8 (1090–3159)	45	3996 (1737–12,382)	56	<0.001
**Urinary indices**							
Urine protein:creatinine	0.81 (0.42–1.11)	81	0.81 (0.52–1.15)	34	0.81 (0.41–1.11)	47	0.867
Urine albumin:creatinine	9.52 (5.49–19.12)	65	9.52 (5.69–19.12)	25	9.76 (5.19–19.49)	40	0.861
pH	5 (5–6)	98	6 (5–6)	42	5 (5–6)	56	<0.001
FeNa (%)^c^	0.67 (0.37–1.35)	94	0.57 (0.22–0.91)	40	0.94 (0.47–2.44)	54	0.012
**Oxidative stress markers**							
Plasma F_2_-IsoPs (pg/ml)^b^	41.1 (34.8–48.5)	64	27.8 (23.7–32.7)	29	56.7 (45.3–70.9)	35	<0.001
Plasma IsoFs (pg/ml)^b^	70.6 (56.2–88.7)	64	41.7 (30.2–57.6)	29	109.2 (85.1–140.1)	35	<0.001

All values are median (IQR) unless otherwise specified: ^b^geometric mean (95% CI), ^c^number (%). *P* < 0.05 using student t-test, Mann U Whitney or Fischer’s exact tests; significant in bold. *Abbreviations: AKI* acute kidney injury, *PfHRP2 P falciparum* histidine rich protein 2, *FeNa* fractional excretion of sodium, *F*_*2*_ *-IsoPs* plasma F_2_-isoprostanes, *IsoFs* plasma isofurans

**Table 3:** Outcomes by AKI status on enrolment

Outcome	Total	No AKI	AKI	*P*
	(*n* = 107)	(*n* = 45)	(*n* = 62)	
AKI stage during admission†				
Stage 1 (≥1.5 × baseline)^c^	11 (12)	4 (10)	7 (15)	0.458
Stage 2 (≥2.0–2.9 × baseline)^c^	11 (12)	1 (2)	10 (21)	**0.021**
Stage 3 (≥3.0 × baseline or ≥4 mg/dl)^c^	36 (40)	5 (12)	31 (65)	**<0.001**
Received RRT (%)^c^	32 (30)	3 (7)	29 (47)	**<0.001**
Length of hospital stay (days)	7.6 (5.6–12.9)	5.9 (4.9–8.3)	10.6 (6.6–18.0)	**<0.001**
Death (%)^c^	35 (33)	10 (22)	25 (40)	0.061
Study hours to death (%)^c^	22.5 (12.5–51.0)	40.1 (19.0–115.0)	21.0 (10.0–38.0)	0.074

All values are median (IQR) unless otherwise specified: ^c^number (%). *P* < 0.05 using Mann U Whitney or Fischer’s exact test; significant in bold. †Highest KDIGO stage during admission in those that survived more than 24 h. Abbreviations: AKI = acute kidney injury; RRT = renal replacement therapy

### Cell-free hemoglobin and oxidative stress

**Fig. 1:**
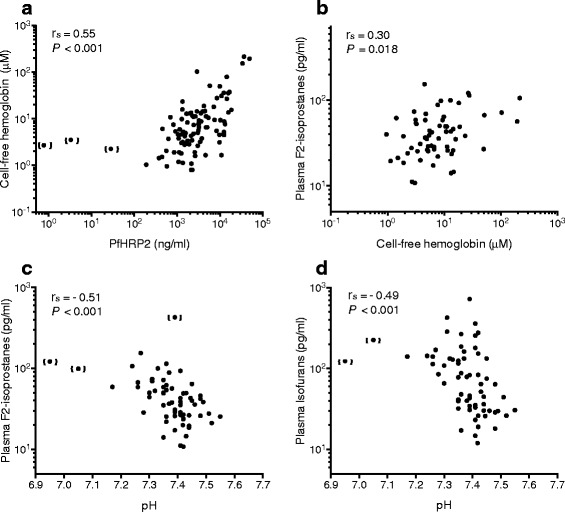
Correlation of oxidative stress markers in severe malaria. **a** Parasite burden, as measured by PfHRP2 concentration, positively correlated with plasma cell-free hemoglobin concentration (*n* = 96), **b** cell-free hemoglobin positively correlated with plasma F_2_-isoprostanes (*n* = 62). Acidemia (venous pH) was inversely correlated with (**c**) plasma F_2_-isoprostanes, and **d** plasma isofurans. All values are from enrolment assessments. *Abbreviations*: PfHRP2, *Plasmodium falciparum* histidine rich protein 2; *r*_*s*_, Spearman’s correlation coefficient

Plasma CFH concentrations were significantly higher in patients with severe malaria (7.0 μM; 95% CI, 5.5-8.7 μM; n=105) compared to those with uncomplicated malaria (4.3 µM; 95% CI, 3.5-5.2 µM; (n=80)Additional file 1: Figure S1; *P* = 0.002) or sepsis (2.5 μM; 95% CI, 1.4–4.3 μM; *n* = 28; *P* < 0.001). Plasma F_2_-IsoPs and IsoFs were also higher in patients with severe malaria compared to uncomplicated malaria (Additional file 1: Figure S1; F_2_-IsoPs, *P* < 0.001; IsoFs, *P* = 0.005). Among those with severe malaria, plasma CFH, F_2_-IsoPs and IsoFs correlated with other measures of hemolysis, including LDH, total bilirubin, and indirect bilirubin. PfHRP2, [26] but not peripheral blood parasitemia, correlated positively with CFH (*r*_*s*_ = 0.55, *P* < 0.001; *r*_*s*_ = 0.18, *P* = 0.08) (Fig. 1A). Similarly, F_2_-IsoPs and IsoFs correlated with PfHRP2 (*r*_*s*_ = 0.34, *P* = 0.008; *r*_*s*_ = 0.31, *P* = 0.017) but neither correlated with parasitemia (*r*_*s*_ = 0.02, *P* = 0.89; *r*_*s*_ = −0.03, *P* = 0.98). CFH weakly correlated with plasma F_2_-IsoPs (*r*_*s*_ = 0.30, *P* = 0.018), as a measure of oxidative stress (Fig. 1B); but the correlation with IsoFs was not significant (*r*_*s*_ = 0.16, *P* = 0.22). Both plasma F_2_-IsoPs and IsoFs were inversely associated with pH (*r*_*s*_ = − 0.51, *P* < 0.001; *r*_*s*_ = − 0.49; *P* < 0.001) (Fig. 1C, D) and positively correlated with base deficit (*r*_*s*_ = 0.59, *P* < 0.001; *r*_*s*_ = 0.63; *P* < 0.001). In a multivariable regression model adjusting for disease severity, CFH and decreasing pH (acidosis) were positive predictors of (log) F_2_-IsoPs (β coefficient 0.13; 95% CI, 0.02 to 0.25; *P* = 0.026; −2.94; 95% CI, −4.24 to −1.64; *P* < 0.001, respectively).

### Cell-free hemoglobin, oxidative stress, and renal function

**Fig. 2:**
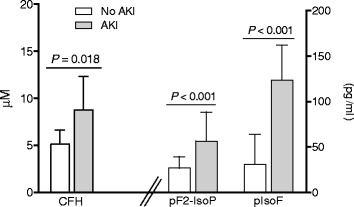
Cell-free hemoglobin, and oxidative stress measures at enrolment in patients with severe malaria by AKI status. Plasma cell-free hemoglobin (*n* = 105), F_2_-isoprostanes (*n* = 64) and isofurans (*n* = 64) were significantly more elevated on enrolment in those with acute kidney injury. Geometric mean and 95% CI are shown. *Abbreviations* *:* AKI, acute kidney injury; CFH, cell-free hemoglobin; pF_2_-IsoP, plasma F_2_-isoprostanes; pIsoF, plasma isofurans

**Fig. 3:**
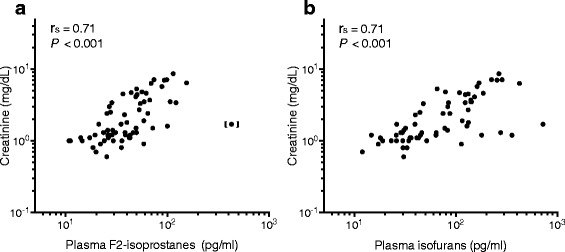
Correlations with creatinine in patients with severe malaria. Creatinine on enrolment positively correlated with (**a**) plasma F_2_-isoprostanes (*n* = 63), and (**b**) plasma isofurans (*n* = 64). *Abbreviations: r*_*s*_, Spearman’s correlation coefficient

In severe malaria, CFH was higher in patients with AKI compared to those without AKI (*P* = 0.018) (Fig. 2; Table 2). CFH in patients with severe malaria-associated AKI was also higher compared to patients with sepsis-related AKI (0.8 μM; 95% CI, 0.1–3.9 μM; *n* = 4; *P* = 0.001). Patients with hemoglobinuria at enrolment had significantly higher CFH (geometric mean: 15.6 μM; 95% CI, 6.9–35.6 μM; *n* = 18), PfHRP2 (median: 10,411 ng/ml; IQR, 2909–14,504 ng/ml; *n* = 18), and serum creatinine (median: 2.9 mg/dl; IQR, 1.3–4.7 mg/dl; *n* = 18) compared to those without hemoglobinuria (CFH: 5.4 μM; 95% CI, 4.3–6.7 μM; *n* = 74; *P* < 0.001; PfHRP2: 2146 ng/ml; IQR, 1266–4216 ng/ml; *n* = 73; *P* = 0.001; creatinine: 1.4 mg/dl; IQR, 1.1–2.7 mg/dl; *n* = 76; *P* = 0.040). In severe malaria, plasma F_2_-IsoPs and IsoFs were significantly higher in patients with AKI compared to those without (both *P <* 0.001) (Fig. 2; Table 2). Enrolment plasma F_2_-IsoPs and IsoFs strongly correlated with enrolment serum creatinine (*r*_*s*_ = 0.71, *P* < 0.001; *r*_*s*_ = 0.71, *P* < 0.001) (Fig. 3A, B) and with the fractional excretion of sodium (*r*_*s*_ = 0.49, *P* < 0.001; *r*_*s*_ = 0.37, *P* = 0.006). The 24-h urine F_2_-IsoP excretion concentration was lower in the AKI group (median: 0.9 ng; IQR, 0.6–2.4 ng; *n* = 9) compared to those without AKI (2.9 ng; IQR, 1.1–4.0 ng; *n* = 11; *P =* 0.037). However, the 24-h urine IsoF excretion concentration was similar between groups (AKI median: 9.7 ng; IQR, 8.1–15.2 ng; *n* = 9; No AKI: 9.2 ng; IQR, 4.3–53.2 ng; *n* = 12; *P* = 0.72).

In patients without AKI on enrolment but subsequently developing AKI, initial plasma IsoFs were higher compared to those who did not develop AKI (geometric mean: 81.6 pg/ml; 95% CI, 28.6–232.9 pg/ml versus 35.0 pg/ml; 95% CI, 25.6–48.0 pg/ml; *P =* 0.027). Furthermore, in this group (excluding 3/10 patients who received hemodialysis) peak plasma concentrations of F_2_-IsoPs and IsoFs at 24 h were higher than in patients not developing AKI (F_2_-IsoPs: 43.5 pg/ml; 95% CI 26.4–71.8 pg/ml; versus 24.8 pg/ml; 95% CI 19.1–32.1 pg/ml; *P* = 0.020; IsoF: geometric mean 220.0 pg/ml; 95% CI, 52.9–914.8 pg/ml versus 48.0 pg/ml; 95% CI, 32.8–70.2 pg/ml; *P* = 0.003). The peak creatinine in the former group was reached at 48 h (median: 4.5 mg/dl; IQR, 1.1–4.6 mg/dl).

Plasma F_2_-IsoPs and IsoFs on enrolment, prior to hemodialysis, were higher among those who received hemodialysis (F_2_-IsoPs: 58 pg/ml; 95% CI, 46–72 pg/ml; *n* = 19; versus 36 pg/ml; 95% CI, 29–44 pg/ml; *n* = 45; *P* = 0.006; IsoFs: 131 pg/ml; 95% CI, 99–173 pg/ml; *n* = 19; versus 54 pg/ml; 95% CI, 41–71 pg/ml; *n* = 45; *P <* 0.002). Plasma CFH at enrolment was not higher in those who received hemodialysis (CFH: 9.0 μM; 95% CI, 5.2–15.4 μM; *n* = 31; versus 6.2 μM; 95% CI, 4.9–7.9 μM; *n* = 74; *P* = 0.15).

### Red cell deformability, renal function and oxidative stress

**Fig. 4:**
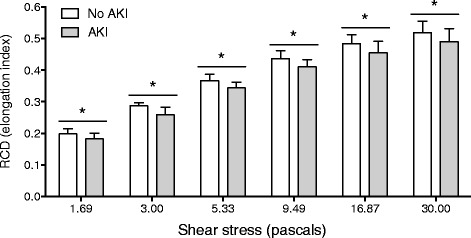
Red cell deformability at enrolment in patients with severe malaria by AKI status. Red cell deformability at shear stresses from 1.69 to 30.0 Pa were significantly lower in the AKI group (*n* = 51) compared to the no AKI group (*n* = 33). *Abbreviations*: AKI, acute kidney injury; RCD, red cell deformability. Asterisks represent *P* < 0.05 significance

RCD at enrolment was lower at shear stresses between 1.69 and 30.00 Pa in patients with AKI on enrolment compared to those without AKI (Figure 4). Decreased RCD correlated with higher creatinine at all these shear stresses and most strongly at 9.49 Pa (*r*_*s*_ = − 0.31, *P* = 0.019). RCD was also lower at shear stresses between 1.69 and 9.49 Pa in patients who subsequently required hemodialysis (all *P* < 0.05). RCD at low shear stress was inversely associated with plasma F_2_-IsoPs (0.3 Pa: *r*_*s*_ = − 0.46, *P =* 0.003; *n* = 39) but not with IsoFs (1.69 Pa: *r*_*s*_ = − 0.30, *P =* 0.062; *n* = 40).

### Predictors of renal function

Since plasma F_2_-IsoPs and IsoFs were collinear, two different multivariable mixed effects models were considered to assess the associations of each of them with change in serum creatinine over time (Table 4, F_2_-IsoP model and IsoF model, respectively). There was a significant interaction between (log)CFH and time in both multivariable models, *P* = 0.010 and *P* = 0.012 for the model that included (log)F_2_-IsoPs and (log)IsoFs, respectively (Table 4). The effect of enrolment CFH on creatinine had a lag time. In the model with F_2_-IsoPs, increased enrolment plasma F_2_-IsoPs, PfHRP2 and decreased red cell deformability were also independently associated with increasing serum creatinine over the first 72 h of admission. In the model with IsoFs, increased enrolment plasma IsoFs, and PfHRP2 were independently associated with an increase in serum creatinine over 72 h. In a logistic regression model adjusted for disease severity, plasma F_2_-IsoPs (OR = 7.37, 95% CI, 1.86–29.23) was independently associated with the need for hemodialysis during admission, but RCD was not (overall model fit *R* ^*2*^ = 0.20; Table 5). In the logistic regression model with IsoFs (rather than F_2_-IsoPs) as independent variable, elevated IsoFs (OR = 5.5, 95% CI, 1.76–17.24) at enrolment was also independently associated with the subsequent need for hemodialysis, but RCD and CFH were not (overall model fit *R* ^*2*^ = 0.41; Table 5).

**Table 4:** Association of variables with change in absolute creatinine over 72 h in patients with severe malaria

	Univariable analysis		Multivariable F_2_-IsoP model		Multivariable IsoF model	
Variable	β (95% CI)^a^	*P*	β (95% CI)^a^	*P*	β (95% CI)^a^	*P*
Log CFH^§^	0.27 (−0.19 to 0.74)	0.249	---	---	---	---
Log F_2_-IsoP#	2.35 (1.27 to 3.43)	*<0.001*	3.01 (1.92 to 4.12)	*<0.001*	---	---
Log IsoF	2.06 (1.46 to 2.65)	*<0.001*	---	---	1.83 (1.26 to 2.40)	*<0.001*
LogPfHRP2	0.61 (0.25 to 0.98)	*0.001*	0.70 (0.02 to 1.38)	*0.045*	0.69 (0.05 to 1.32)	*0.034*
RCD at SS 1.69 Pa	−1.67 (−14.93 to 11.59)	0.805	---	---	---	---
RCD at SS 9.49 Pa	−6.42 (−15.10 to 2.26)	0.147	−4.26 (−7.86 to −0.67)	*0.020*	−2.46 (−5.92 to 1.00)	0.163
Age	0.01 (−0.03 to 0.05)	0.624	---	---	---	---
SBP	0.03 (−0.01 to 0.06)	0.094			---	---
Visit (time)	0.65 (0.52 to 0.78)	*<0.001*	---	---	---	---
Log CFH × Log F_2_-IsoP	0.31 (−0.19 to 0.86)	0.221	---	---	---	---
Log CFH × Log IsoF	0.62 (0.27 to 0.98)	*0.001*	---	---	---	---
Log F_2_-IsoP × visit	0.85 (0.55 to 1.14)	*<0.001*	---	---	---	---
Log IsoF × visit	0.62 (0.47 to 0.76)	*<0.001*	---	---	---	---
Log CFH × visit	0.10 (−0.10 to 0.21)	0.076	0.21 (0.05 to 0.36)	*0.010*	0.20 (0.04 to 0.36)	*0.012*

^a^Regression coefficient (β) with 95% confidence intervals (CIs) showing the estimated decrease in creatinine predicted by a 1-U change in the independent (predictor) variables*.* Interaction terms improved the model fit. *Abbreviations*: *CFH* cell free hemoglobin, *F*_*2*_ *-IsoP* plasma F_2_-isoprostanes, *IsoF* plasma isofurans, *CFH × F*_*2*_ *-IsoP* cell-free hemoglobin and plasma F_2_-isoprostane interaction term, *CFH × IsoF* cell-free hemoglobin and plasma isofuran interaction term, *PfHRP2 P. falciparum* histidine rich protein 2, *RCD* red cell deformability at shear stress 1.69 and 9.49 Pa. # Plasma IsoFs collinear with plasma F_2_- IsoPs. P-values in italics denote statistical significance

**Table 5:** Association of variables with subsequent hemodialysis requirement in patients with severe malaria

	Univariable analysis		Multivariable F_2_-IsoP model		Multivariable IsoF model	
Variable	OR (95% CI)^a^	*P*	OR (95% CI)^a^	*P*	OR (95% CI)^a^	*P*
CFH	1.01 (0.99 to 1.03)	0.063	---	---	1.06 (0.98 to 1.14)	0.156
Log F_2_-IsoP#	3.45 (1.30 to 9.16)	*0.013*	7.37 (1.86 to 29.23)	*0.005*	---	---
Log IsoF	3.48 (1.62 to 7.49)	*0.001*	---	---	5.50 (1.76 to 17.24)	*0.003*
LogPfHRP2	1.92 (1.27 to 2.91)	*0.002*	---	---	---	---
LogRCD at SS 1.69 Pa	0.29 (0.08 to 1.09)	0.066	---	---	---	---
LogRCD at SS 9.49 Pa	0.10 (0.01 to 0.75)	*0.025*	0.057 (0.002 to 1.33)	0.075	0.031 (0.001 to 1.44)	0.076
Age	1.00 (0.97 to 1.03)	0.940	---	---	---	---
SBP	1.02 (0.99 to 1.04)	0.260			---	---
Number of severity criteria	1.90 (1.38 to 2.62)	*<0.001*	---	---	---	---
GCS	0.95 (0.85 to 1.07)	0.393	---	---	---	---
LogLactate	1.22 (0.64 to 2.30)	0.548	---	---	---	---

^a^Odds ratio (OR) with 95% confidence intervals (CIs) showing the hemodialysis requirement predicted by a 1-U change in the independent (predictor) variables. A backward stepwise multivariable model included all variables in univariable analyses which were removed on the basis of *P ≥* 0.05. *Abbreviations*: *CFH* cell free hemoglobin, *F*_*2*_ *-IsoP* plasma F_2_-isoprostanes, *IsoF* plasma isofurans, *PfHRP2 P. falciparum* histidine rich protein 2, *RCD* red cell deformability at shear stress 1.69 and 9.49 Pa, *SBP* systolic blood pressure, *GCS* Glasgow Coma Score. # Plasma IsoFs collinear with plasma F_2_- IsoPs. P-values in italics denote statistical significance

### Cell-free hemoglobin, oxidative stress and survival

Those who died had higher enrolment plasma F_2_-IsoPs (geometric mean: 55 pg/ml; 95%CI, 38–79; *n* = 21) and IsoFs (geometric mean: 97 pg/ml; 95%CI, 64–145; *n* = 21) compared to survivors (F_2_-IsoPs: 36 pg/ml; 95%CI, 30–42; *n* = 43; *P* = 0.014; IsoFs: 61 pg/ml; 95%CI, 46–80; *n* = 43; *P =* 0.054). There was no difference in CFH between those who died and survived (*P* = 0.143).

## Discussion

In this study, plasma cell-free hemoglobin and oxidative stress markers (F_2_-IsoPs and IsoFs) were strongly associated with the presence of AKI on admission, and oxidative stress markers predicted subsequent creatinine elevation and hemodialysis requirement during admission in adult patients with severe falciparum malaria.

Earlier studies have shown that urine F_2_-IsoPs and other urine and plasma oxidative stress markers are elevated in severe malaria compared to uncomplicated malaria [18–21]. CFH-mediated oxidative stress has been shown to contribute to AKI in diseases and medical procedures inducing hemolysis [15–17, 35, 36]. In malaria, intravascular hemolysis involves both parasitized and non-parasitized red blood cells [2]. In the current study, plasma CFH was associated with higher parasite burden, as measured by PfHRP2, consistent with a previous study [1]. This suggests that the hemoglobin released at schizont rupture, the end of the 48-h intra-erythrocytic lifecycle, contributes to plasma CFH concentration.

Redox cycling of hemoglobin forms a radical species that can initiate lipid peroxidation of arachidonic acid to generate F_2_-IsoPs and IsoFs [11, 12, 37]. In this study, CFH concentrations correlated with increased levels of plasma F_2_-IsoPs, a marker of oxidative stress. Several factors influence the generation of lipid peroxidation markers. The oxidative capacity of plasma CFH is largely dependent on its redox state and the fate of the heme moiety. Heme is water-insoluble; it binds to hemopexin, albumin, lipoproteins, and cell membranes [38], and its oxidative capacity differs considerably between these fractions. In addition, other sources of free radicals, such as those created during phagocytic oxidative burst, may initiate lipid peroxidation to generate plasma F_2_-IsoPs and IsoFs. Indeed, renal histopathology of AKI in severe malaria shows accumulation of host monocytes (in addition to parasitized red blood cells) in the renal microvasculature [39]. Heme-containing myoglobin concentrations are also increased in severe malaria, although to a much lesser extent than CFH [1]. The oxidative capacity of myoglobin increases at low pH because of increased pseudoperoxidase activity [10]. This could also apply to CFH, given that both plasma F_2_-IsoPs and IsoFs were associated with reduced venous blood pH in the current study.

F_2_-IsoPs are potent renal vasoconstrictors, which reduce renal blood flow and GFR [12]. In this study, concentrations of plasma CFH, F_2_-IsoPs, and IsoFs at enrolment were all higher in patients with AKI, whereas F_2_-IsoPs and IsoFs correlated with the fractional excretion of sodium (an indirect measure of renal tubular injury) and were independently associated with increased creatinine over 72 h and the subsequent requirement for hemodialysis. A common problem of AKI biomarkers is that their concentrations can be increased as a result of renal dysfunction, rather than be a cause of it. Indeed, the lower 24-h urine F_2_-IsoP excretion concentrations in patients with established AKI suggest that hemoglobin dimers and/or F_2_-IsoPs are not filtered well at low creatinine clearances. However, the group of patients without AKI on enrolment that subsequently developed AKI also showed elevated enrolment plasma IsoF concentrations. This was then followed by concomitant increases in 24 h plasma F_2_-IsoPs and IsoF with a subsequent peak in serum creatinine at 48 h. This time course suggests a role of plasma F_2_-IsoPs and IsoFs in the pathogenesis of AKI. The delayed effect of CFH-mediated oxidative stress on renal function is also suggested by the multivariable mixed effects model, in which time modified the effect of enrolment CFH on creatinine, where the slope of change in creatinine increased with time.

Plasma F_2_-IsoPs were also associated with reduced RBC deformability. Red cell rigidity has been well-described in severe malaria [27]. It is thought to be caused by oxidative damage to RBC membranes, and contribute to microvascular flow obstruction [27, 28]. RBC membranes are rich in arachidonic acid. Heme-mediated lipid peroxidation of RBC membranes could be an important cause of reduced RCD, and a significant source of plasma F_2_-IsoPs and IsoFs in severe malaria. RCD at low shear stresses is mainly determined by membrane properties, whereas at high shear stresses surface-to-volume relationships become more important [40, 41]. In this study, the correlation between plasma F_2_-IsoPs and decreased RCD was strongest at low shear stress, suggesting RBC membrane damage. In the current study, RCD was lower in patients with AKI, inversely correlated with serum creatinine, and independently associated with an increase in serum creatinine over 72 h. Reduced RCD could further compromise renal medullary perfusion concomitant with F_2_-IsoP- and IsoF-induced vasoconstriction and obstructing microvascular sequestered parasitized red blood cells, all contributing to renal tubular damage [39].

This study has some limitations. The sample size of this detailed study was relatively small. Additional biomarkers of AKI were not assessed. However, both changes in serum creatinine and hemodialysis are well established indicators of kidney dysfunction and injury. Living or post-mortem renal biopsy as a standard to evaluate the underlying kidney pathology was not ethical or feasible in this study. Quantification of plasma and urine hemoglobin oxidation states was not performed. However, studies have shown that methemoglobin is the predominant form in patients with malaria and blackwater fever [42]. Myoglobin as an alternative source of heme-mediated oxidative damage was not assessed, but other studies have shown a 100-fold less increase in plasma myoglobin compared to CFH in patients with severe malaria [1].

## Conclusions

In summary, the findings of this study suggest that CFH and systemic F_2_-IsoPs and IsoFs contribute to the pathogenesis of AKI in severe malaria and these markers of oxidative stress are associated with need for hemodialysis and in-hospital mortality. CFH can have a direct oxidative damaging effect on renal tubules. In addition, CFH induced lipid peroxidation may have a dual effect in the mechanism of AKI in malaria. Firstly, the lipid peroxidation metabolites, plasma F_2_-IsoPs, may cause direct renal vasoconstriction. Secondly, lipid peroxidation of RBC membranes may result in reduced RCD, which in turn could exacerbate ischemia in the renal medulla.

Therapies targeted at reducing hemoprotein-mediated oxidative stress have been shown to improve renal function and mortality [9, 17, 35]. Treatments that reduce CFH levels or CFH-mediated oxidative stress, such as haptoglobin or paracetamol respectively, may have potential as an adjunctive therapy to improve kidney function and survival in severe malaria.

## Additional file

Additional file 1: Figure S1. Supplement to Plewes K, Kingston HWF, Ghose A, et al. Cell-free hemoglobin mediated oxidative stress is associated with acute kidney injury and renal replacement therapy in severe falciparum malaria: an observational study. Cell-free hemoglobin, and oxidative stress measures at enrolment stratified by malaria severity. Plasma cell-free hemoglobin (*n* = 185), F_2_-isoprostanes (*n* = 82) and isofurans (*n* = 82) were significantly more elevated on enrolment in those with severe malaria compared to uncomplicated malaria. Geometric mean and 95% CIs shown. *Abbreviations* ***:*** AKI, acute kidney injury; CFH, cell-free hemoglobin; pF_2_-IsoP, plasma F_2_-isoprostanes; pIsoF, plasma isofurans. (DOCX 1707 kb)

## Abbreviations

AKI: acute kidney injury
CFH: cell-free hemoglobin
F_2_-IsoPs: F_2_-isoprostanes
GFR: glomerular filtration rate
IsoF: Isofurans
KDIGO: Kidney Diseases Improving Global Outcomes
PfHRP2: *Plasmodium falciparum* histidine rich protein 2
RBC: red blood cell
RCD: red cell deformability
RRT: renal replacement therapy
