# Supplementary material for: Cell-free hemoglobin mediated oxidative stress is associated with acute kidney injury and renal replacement therapy in severe falciparum malaria: an observational study
Source: BMC Infect Dis. 2017 Apr 27;17:313. doi: 10.1186/s12879-017-2373-1 (PMC5408414; doi:10.1186/s12879-017-2373-1)
Supplement: Additional file 1: Figure S1. — Supplement to Plewes K, Kingston HWF, Ghose A, et al. Cell-free hemoglobin mediated oxidative stress is associated with acute kidney injury and renal replacement therapy in severe falciparum malaria: an observational study. Cell-free hemoglobin, and oxidative stress measures at enrolment stratified by malaria severity. Plasma cell-free hemoglobin (n = 185), F2-isoprostanes (n = 82) and isofurans (n = 82) were significantly more elevated on enrolment in those with severe malaria compared to uncomplicated malaria. Geometric mean and 95% CIs shown. Abbreviations : AKI, acute kidney injury; CFH, cell-free hemoglobin; pF2-IsoP, plasma F2-isoprostanes; pIsoF, plasma isofurans. (DOCX 1707 kb) [file 12879_2017_2373_MOESM1_ESM.docx]

**Supplementary file**

Supplement to Plewes K, Kingston HWF, Ghose A, et al. Cell-free hemoglobin mediated oxidative stress is associated with acute kidney injury and renal replacement therapy in severe falciparum malaria: an observational study

**Supplementary figure**


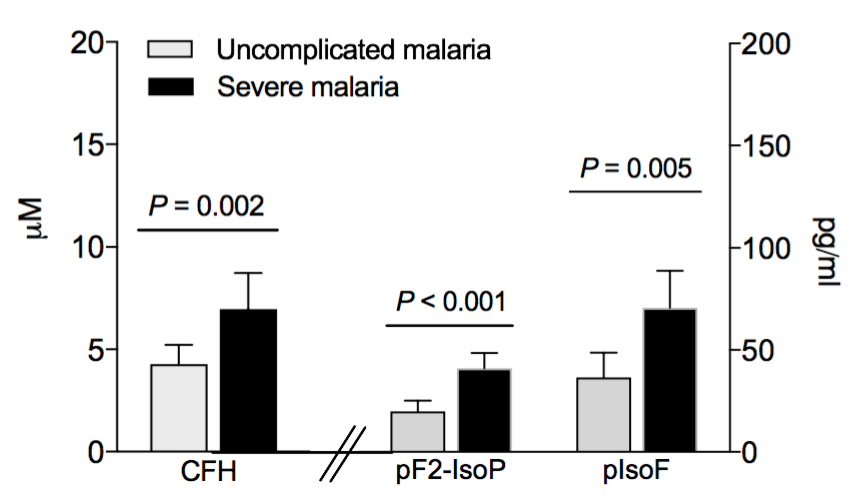


**Supplementary Figure 1.** Cell-free hemoglobin, and oxidative stress measures at enrolment stratified by malaria severity. Plasma cell-free hemoglobin (n=185), F_2_-isoprostanes (n=82) and isofurans (n=82) were significantly more elevated on enrolment in those with severe malaria compared to uncomplicated malaria. Geometric mean and 95% CIs shown. *Abbreviations****:*** AKI, acute kidney injury; CFH, cell-free hemoglobin; pF_2_-IsoP, plasma F_2_-isoprostanes; pIsoF, plasma isofurans.
